# St. Luke’s Hospital

**Published:** 1872-05

**Authors:** Jno. E. Owens


ARTICLE III. — *St. Luke's Hospital.* Excision of first phalanx of right thumb. Excision of heads of bones composing the second phalangeal articulation of the forefinger. Urinary calculus; lithotomy; disorganization of kidneys; vesico-perineal fistule; death. (Under the care of JNO. E. OWENS, M.D.)

W. N., aged 28 years, sustained a contusion of the right thumb. The case terminated in necrosis and caries of the first phalanx. Upon admission, two weeks after the accident, the thumb was very much swollen, purplish, and discharging pus from three or four fistulous orifices. Two weeks subsequent to admission, the first phalanx, in its entirety, was removed through a straight incision on the dorsum of the thumb. At the end of nine weeks from the date of admission, the wounds had completely healed, with the following result:

The thumb was straight; distance from styloid process of radius to the end of the thumb was four inches, being one inch shorter than that of the other hand. The patient is able to approximate the end of the thumb to the ends of the fore and middle fingers. He will be able to perform the same with the ring and little fingers. He has the power of flexion in a very slight degree at the new joint. The muscles, after the operation, gradually approximated the distal phalanx and the meta-carpal bone.

The thumb is of special importance, and if only a single finger is left to oppose it or any part of it, great use may still be made of the hand.

M. G., aged 25 years, was admitted with a felon, and caries of the articulating surfaces of the bones composing the second phalangeal joint. On the day of admission, a straight incision was made in the palmar aspect of the forefinger, the diseased portions of the bone cut off with the bone forceps, and the wound closed with adhesive plaster. Three weeks after the operation the finger had recovered, with the power of flexion and extension.

A. P., aged 24 years, Canadian, married, was admitted with the usual subjective symptoms of stone in the bladder, viz., pain in the glans penis, at the lower part of the back, in the perineum, and over the pubis, the pain being more severe at the end of micturition; frequency of micturition, (55 times during the first day in the hospital); occasional hæmaturia; sudden stoppage of the passage of water. The urine was not albuminous. The symptoms were increased by exercise, but a carriage ride increased them to a greater degree. At the age of 12 years, the patient remembers to have had frequent and painful micturition, from which symptoms he suffered more or less annoyance to the date of admission to the hospital. About four years ago he passed a stone, the size of a pea and frequently saw sand in the bottom of the chamber, and occasionally blood. The patient was thin, sallow, irritable, and hyperæsthetic. So excessive was the irritability of the urethra, that the gentlest instrumentation was followed by urethral fever. A rigor was excited merely by manipulating the glans penis. Only once did we succeed in the introduction of a sound without the use of an anæsthetic, and then the patient suffered most intensely.

A week after admission, the unilateral operation was performed and a phosphatic stone extracted, $1\frac{3}{4}$ in. long, $1\frac{1}{8}$ in. wide, $\frac{3}{4}$ in. thick, $3\frac{1}{16}$ in. circumference, and weighing dr. vj, grs. xij. Morph.

sulph., gr. ss, injected beneath the skin of the shoulder, produced sleep in five minutes. Suffice it to say, that, although the local symptoms after a time subsided, the patient loathed the very sight of nourishment, and that, too, in spite of the administration of tonics and the exhibition of the most tempting food. Two weeks after the operation a diffuse abscess in the right arm was opened, and three weeks after, slight œdema of the legs appeared. Rheumatic pains of right shoulder and left leg supervened, and, although not severe, caused more or less annoyance to the patient.

Four weeks subsequent to the operation, the patient died. Diarrhœa, with involuntary discharges, and coma, closed the scene. The wound in the perineum never entirely closed.

*Autopsy.* Chronic inflammation and thickening of the bladder; a vesico-urethral fistule, size of a No. 3 catheter; right kidney had degenerated into a membranous sack, and the secreting structure of the left was almost entirely disorganized. The cause of death in this case was organic disease of the kidneys, and it is a matter of surprise that a patient can live so long with such extensive disorganization of these great emunctories.

There was, in this case, a prominent symptom which I did not at the time fully appreciate, viz., the general hyperæsthesia and the severe urethral fever or rigor which constantly followed slight urethral irritation. The patient to the time of his death was in a constant state of *noli me tangere.* Many cases, not only of diseases of the urinary organs, but also of surgical affections generally, in which this general hyperæsthesia was a prominent symptom, have taught me to look upon this symptom as indicative, in many cases at least, of organic renal disease. I can call to mind at this time a significant case. The patient, a woman, had chronic inflammation of the knee joint. General hyperæsthesia was a prominent symptom, on which account we delayed excision of the joint. A short time afterwards, the patient dying, the kidneys were found to be nearly as much disorganized as those in the former case.

Organic disease of the kidneys, more than that of any other organ, is the cause of fatal results, after surgical operations, often in their nature trivial.

